# Supplementary material for: Long-Term Periodic and Conditional Survival Trends in Prostate, Testicular, and Penile Cancers in the Nordic Countries, Marking Timing of Improvements
Source: Cancers (Basel). 2023 Aug 25;15(17):4261. doi: 10.3390/cancers15174261 (PMC10486399; doi:10.3390/cancers15174261)
Supplement: Supplementary file 1 [file cancers-15-04261-s001.zip › cancers-2505361-supplementary.pdf]

Periodic and conditional survival trends in prostate, testicular and penile cancers in the nordic countries: marking timing of improvements

Filip Tichanek, Asta Försti, Akseli Hemminki, Otto Hemminki, Kari Hemminki

Supplementary information

Supplementary Table S1. 1-year (left part) and 5-years (right part) relative survival [95% confidence interval] in male-associated cancers from 1971 to 2020, across 4 Nordic countries. Cancer localizations include: (a) testis, (b) prostate, (c) penis. *Significant increase between the marked and the next period (95% confidence intervals do not touch estimates in another period).

1-year survival					5-years survival			
(a) testis	Denmark	Finland	Norway	Sweden	Denmark	Finland	Norway	Sweden
1971-1975	82.6 [78.5-86.8]*	75.0 [67.5-83.4]	82.0 [76.3-88.0]*	83.9 [80.2-87.8]	66.4 [60.6-72.8]*	56.5 [47.7-66.8]	60.6 [53.4-68.7]*	71.7 [66.1-77.8]
1976-1980	87.8 [83.9-91.8]	79.8 [73.0-87.3]	89.9 [84.3-96.0]	84.8 [81.3-88.3]*	80.6 [75.8-85.8]	63.9 [54.7-74.5]*	79.2 [70.5-88.9]	76.3 [71.8-81.0]*
1981-1985	91.2 [87.7-94.9]	85.1 [79.3-91.3]	90.9 [85.8-96.3]	94.9 [92.4-97.6]	85.5 [80.6-90.8]	76.4 [68.7-85.0]	82.3 [74.4-91.1]	91.1 [86.2-96.3]
1986-1990	92.0 [88.7-95.4]	89.3 [83.0-96.2]	92.7 [88.6-96.9]	94.7 [91.9-97.7]	85.9 [80.6-91.5]*	82.2 [73.3-92.3]	89.3 [83.7-95.4]	95.1 [90.9-99.6]
1991-1995	93.1 [90.1-96.3]	89.9 [83.4-97.0]	91.3 [86.6-96.3]*	94.1 [91.1-97.2]	92.1 [87.7-96.6]	85.9 [77.3-95.5]	88.2 [82.2-94.7]	90.9 [86.3-95.6]
1996-2000	94.9 [91.4-98.4]	91.4 [85.5-97.7]*	97.5 [94.7-100.5]	95.8 [92.9-98.8]	92.5 [87.4-97.9]	88.3 [80.7-96.6]	96.3 [91.5-101.4]	91.1 [85.6-96.9]
2001-2005	94.1 [90.6-97.8]	97.8 [94.3-101.5]	93.9 [90.2-97.8]	95.2 [91.9-98.6]	95.1 [91.2-99.2]	94.4 [87.2-102.3]	91.9 [86.9-97.1]	94.7 [89.8-99.9]
2006-2010	97.2 [94.7-99.6]	94.9 [90.6-99.5]	94.7 [90.9-98.8]	97.5 [95.4-99.6]	97.3 [93.9-100.9]	93.7 [87.8-100.0]	92.2 [87.1-97.6]	96.6 [93.2-100.1]
2011-2015	97.9 [96.0-99.8]	91.3 [86.6-96.3]	95.9 [93.0-98.9]	97.2 [95.2-99.2]	97.8 [94.8-101.0]	88.7 [82.5-95.4]	96.1 [92.3-100.0]	98.9 [96.1-101.7]
2016-2020	97.7 [96.0-99.6]	96.4 [93.4-99.6]	97.7 [95.4-100.0]	97.5 [95.6-99.5]	95.3 [92.1-98.6]	93.5 [88.5-98.7]	96.7 [93.0-100.6]	98.8 [96.0-101.6]
(b) prostate								
1971-1975	77.8 [75.3-80.4]*	82.3 [79.0-85.8]	84.6 [82.5-86.8]*	83.8 [82.8-84.9]*	39.0 [36.1-42.2]	46.3 [42.9-50.0]	51.0 [48.1-54.2]	53.0 [51.1-55.0]*
1976-1980	80.9 [79.2-82.6]	85.6 [83.8-87.5]*	87.3 [85.4-89.2]	87.9 [86.6-89.3]*	41.0 [38.1-44.2]	49.3 [45.8-53.0]*	52.1 [48.7-55.8]*	57.6 [55.4-59.8]
1981-1985	82.0 [79.7-84.2]	87.9 [86.0-89.9]	87.9 [85.6-90.3]	91.5 [90.5-92.6]*	42.8 [39.9-45.8]	53.5 [50.3-56.8]*	56.0 [52.7-59.4]	58.6 [56.5-60.9]*
1986-1990	82.9 [80.0-86.0]	89.1 [87.1-91.1]*	87.9 [85.8-90.0]*	93.3 [92.5-94.0]*	39.3 [36.1-42.6]	57.2 [54.0-60.6]*	54.9 [52.0-57.9]*	64.1 [62.2-66.0]*
1991-1995	84.2 [82.4-86.0]	92.3 [91.0-93.7]*	91.4 [89.7-93.1]*	94.3 [93.5-95.0]*	39.3 [36.6-42.2]*	65.8 [63.2-68.5]*	63.7 [61.4-66.1]*	68.2 [66.5-70.0]*
1996-2000	86.0 [83.9-88.1]*	95.5 [94.6-96.4]*	94.9 [94.2-95.6]*	96.3 [95.9-96.7]*	51.0 [48.5-53.6]*	80.4 [78.7-82.1]*	77.6 [76.1-79.1]*	78.6 [77.5-79.8]*
2001-2005	92.6 [91.5-93.8]*	97.8 [97.4-98.2]*	96.5 [96.1-96.9]*	97.9 [97.6-98.2]*	71.8 [70.0-73.7]*	91.1 [90.2-92.0]*	84.1 [83.0-85.2]*	88.2 [87.5-89.0]*
2006-2010	97.3 [96.9-97.7]*	98.6 [98.3-98.9]	97.9 [97.5-98.3]*	98.4 [98.2-98.7]*	86.4 [85.4-87.4]*	93.5 [92.7-94.3]	91.0 [90.1-91.8]*	91.8 [91.2-92.3]*
2011-2015	98.2 [97.9-98.5]*	98.6 [98.3-98.9]	99.4 [99.1-99.6]	98.9 [98.7-99.1]*	89.9 [89.0-90.8]	93.1 [92.3-93.9]	94.7 [94.0-95.4]	93.8 [93.3-94.3]*
2016-2020	98.6 [98.3-98.9]	98.8 [98.4-99.1]	99.3 [99.1-99.6]	99.2 [99.1-99.4]	90.1 [89.3-91.0]	93.6 [92.8-94.4]	94.9 [94.2-95.6]	94.9 [94.4-95.4]
(c) penis								
1971-1975	83.0 [76.8-89.6]	84.1 [73.4-96.4]	87.9 [80.9-95.5]	88.1 [83.8-92.6]	64.0 [54.1-75.6]	43.2 [28.9-64.4]	75.7 [63.4-90.2]	75.9 [68.1-84.4]
1976-1980	88.3 [83.2-93.7]	78.6 [66.1-93.4]	88.2 [81.4-95.5]	89.6 [85.4-94.0]	75.4 [66.5-85.6]	60.9 [45.1-82.1]	79.4 [67.0-94.1]	74.0 [66.3-82.7]
1981-1985	85.2 [79.6-91.2]	84.4 [74.6-95.5]	90.6 [84.9-96.7]	88.8 [84.9-93.0]	73.7 [65.0-83.7]	62.6 [47.6-82.4]	74.7 [64.2-86.9]	71.4 [64.3-79.3]
1986-1990	81.8 [76.0-88.0]*	87.7 [77.6-99.1]	93.5 [88.0-99.3]	89.9 [85.9-94.1]	67.1 [58.6-76.8]	68.2 [49.7-93.8]	77.3 [65.6-91.1]	74.4 [67.3-82.3]
1991-1995	89.1 [84.1-94.5]	80.3 [71.2-90.7]	87.8 [81.8-94.2]	85.4 [81.3-89.7]	70.0 [61.1-80.1]	68.6 [55.8-84.4]	69.6 [59.8-80.9]	71.0 [64.5-78.0]
1996-2000	85.5 [80.3-91.0]	81.9 [73.0-92.0]	90.1 [84.5-96.1]	86.5 [82.5-90.8]	73.4 [65.0-83.0]	62.9 [50.8-77.9]	72.0 [61.4-84.4]	71.3 [64.8-78.4]
2001-2005	85.5 [80.5-90.8]	82.8 [74.2-92.4]	89.1 [84.2-94.2]	86.6 [83.1-90.2]*	81.5 [73.6-90.2]	65.3 [52.9-80.6]	79.2 [71.1-88.1]	69.2 [63.6-75.3]*
2006-2010	85.7 [81.1-90.5]	85.1 [78.8-91.9]	90.2 [85.7-94.9]	91.9 [89.0-94.9]	75.1 [68.1-82.8]*	69.4 [59.9-80.3]	79.4 [72.0-87.6]	78.5 [73.1-84.2]
2011-2015	90.1 [86.4-93.8]	87.3 [82.4-92.4]	92.0 [88.2-96.0]	88.6 [85.5-91.8]	83.0 [76.6-89.9]	68.2 [60.7-76.6]	73.4 [66.3-81.3]	75.2 [70.4-80.3]
2016-2020	91.5 [88.2-95.0]	87.3 [82.3-92.5]	91.4 [87.9-94.9]	89.4 [86.8-92.1]	85.7 [80.0-91.7]	68.6 [61.2-76.9]	76.5 [70.3-83.2]	77.1 [72.8-81.6]

Supplementary Table S2. 5/1-year (4-years conditional) survival (left), and difference between 1-year and 5-years survival (right) in male-associated cancers from 1971 to 2020, across 4 Nordic countries. Cancer localizations include: a) testis, (b) prostate, (c) penis.

5-years/1-year (4-years conditional) survival					1-year -- 5-years relative survival difference			
(a) testis	Denmark	Finland	Norway	Sweden	Denmark	Finland	Norway	Sweden
1971-1975	80.4	75.3	73.9	85.5	16.2	18.5	21.4	12.2
1976-1980	91.8	80.1	88.1	90.0	7.2	15.9	10.7	8.5
1981-1985	93.8	89.8	90.5	96.0	5.7	8.7	8.6	3.8
1986-1990	93.4	92.0	96.3	100.4	6.1	7.1	3.4	-0.4
1991-1995	98.9	95.6	96.6	96.6	1.0	4.0	3.1	3.2
1996-2000	97.5	96.6	98.8	95.1	2.4	3.1	1.2	4.7
2001-2005	101.1	96.5	97.9	99.5	-1.0	3.4	2.0	0.5
2006-2010	100.1	98.7	97.4	99.1	-0.1	1.2	2.5	0.9
2011-2015	99.9	97.2	100.2	101.7	0.1	2.6	-0.2	-1.7
2016-2020	97.5	97.0	99.0	101.3	2.4	2.9	1.0	-1.3
(b) prostate								
1971-1975	50.1	56.3	60.3	63.2	38.8	36.0	33.6	30.8
1976-1980	50.7	57.6	59.7	65.5	39.9	36.3	35.2	30.3
1981-1985	52.2	60.9	63.7	64.0	39.2	34.4	31.9	32.9
1986-1990	47.4	64.2	62.5	68.7	43.6	31.9	33.0	29.2
1991-1995	46.7	71.3	69.7	72.3	44.9	26.5	27.7	26.1
1996-2000	59.3	84.2	81.8	81.6	35.0	15.1	17.3	17.7
2001-2005	77.5	93.1	87.2	90.1	20.8	6.7	12.4	9.7
2006-2010	88.8	94.8	93.0	93.3	10.9	5.1	6.9	6.6
2011-2015	91.5	94.4	95.3	94.8	8.3	5.5	4.7	5.1
2016-2020	91.4	94.7	95.6	95.7	8.5	5.2	4.4	4.3
(c) penis								
1971-1975	77.1	51.4	86.1	86.2	19.0	40.9	12.2	12.2
1976-1980	85.4	77.5	90.0	82.6	12.9	17.7	8.8	15.6
1981-1985	86.5	74.2	82.5	80.4	11.5	21.8	15.9	17.4
1986-1990	82.0	77.8	82.7	82.8	14.7	19.5	16.2	15.5
1991-1995	78.6	85.4	79.3	83.1	19.1	11.7	18.2	14.4
1996-2000	85.8	76.8	79.9	82.4	12.1	19.0	18.1	15.2
2001-2005	95.3	78.9	88.9	79.9	4.0	17.5	9.9	17.4
2006-2010	87.6	81.6	88.0	85.4	10.6	15.7	10.8	13.4
2011-2015	92.1	78.1	79.8	84.9	7.1	19.1	18.6	13.4
2016-2020	93.7	78.6	83.7	86.2	5.8	18.7	14.9	12.3
